# Host Responses and Regulation by NFκB Signaling in the Liver and Liver Epithelial Cells Infected with A Novel Tick-borne Bunyavirus

**DOI:** 10.1038/srep11816

**Published:** 2015-07-02

**Authors:** Qiyu Sun, Cong Jin, Lili Zhu, Mifang Liang, Chuan Li, Carol J. Cardona, Dexin Li, Zheng Xing

**Affiliations:** 1 The State Key Laboratory of Pharmaceutical Biotechnology and Medical School, Nanjing University, Nanjing, China; 2 National Institute for Viral Disease Control and Prevention, Chinese Center for Disease Control and Prevention, Beijing, China; 3 Veterinary and Biomedical Sciences, College of Veterinary Medicine, University of Minnesota, Twin Cities, Saint Paul, MN, USA

## Abstract

Infection in humans by severe fever with thrombocytopenia syndrome virus (SFTSV), a novel bunyavirus transmitted by ticks, is often associated with pronounced liver damage, especially in fatal cases. Little has been known, however, about how liver cells respond to SFTSV and how the response is regulated. In this study we report that proinflammatory cytokines were induced in liver tissues of C57/BL6 mice infected with SFTSV, which may cause tissue necrosis in mice. Human liver epithelial cells were susceptible to SFTSV and antiviral interferon (IFN) and IFN-inducible proteins were induced upon infection. We observed that infection of liver epithelial cells led to significant increases in proinflammatory cytokines and chemokines, including IL-6, RANTES, IP-10, and MIP-3a, which were regulated by NFκB signaling, and the activation of NFκB signaling during infection promoted viral replication in liver epithelial cells. Viral nonstructural protein NSs was inhibitory to the induction of IFN-β, but interestingly, NFκB activation was enhanced in the presence of NSs. Therefore, NSs plays dual roles in the suppression of antiviral IFN-β induction as well as the promotion of proinflammatory responses. Our findings provide the first evidence for elucidating host responses and regulation in liver epithelial cells infected by an emerging bunyavirus.

Severe fever with thrombocytopenia virus (SFTSV)^1^,^2^,^3^is an emerging pathogen causing a febrile syndrome comprising high fever, drastic loss of thrombocytes and leukocytes, and, in severe cases, multi-organ failure^1^. SFTSV belongs to the genus *Phlebovirus* in the family *Bunyaviridae*, contains a negative sense, single-stranded and segmented RNA genome, and can be transmitted by ticks^1^,^4^from animals to humans. Since its emergence in central regions of China around 2007, the virus has spread to over 13 provinces. In addition, there is a high prevalence of SFTSV infection in a broad range of domestic animals including sheep, goats, cattle, pigs, and dogs^5^. Recently, SFTS patients have been reported and SFTSV was isolated from SFTS patients in South Korea and Japan in 2012 and 2013^6^,^7^,^8^, respectively. Meanwhile, a closely related Heartland virus was reported in Missouri, USA, in 2012, from two patients with severe symptoms identical to those in SFTS^9^,^10^,^11^,^12^, making this novel group of phleboviruses a regional or global potential threat to both public health and agriculture.

The genome of SFTSV possesses large (L), medium (M), and small (S) segments, with the L segment encoding viral RNA polymerase and the M encoding the two envelope glycoproteins, G_n_ and G_c_. The S segment is an ambisense RNA of 1744 bases in length encoding a nucleoprotein (NP) and a nonstructural protein (NSs) with open reading frames in sense and complementary RNA, respectively^1^,^3^,^13^. The NSs of SFTSV was found to inhibit the IFN-β and NFκB promoter activities induced by viral infection^14^,^15^. Recently, the interaction of SFTSV NSs and tank binding kinase 1 (TBK1) revealed a novel mechanism for a virus to evade innate immunity by sequestrating TBK1, together with the IKK complex essential in IFN-β induction, into cytoplasmic inclusion bodies formed by NSs^15^. Current data support the hypothesis that the viral inclusion bodies themselves appear to function in support of viral RNA replication as well^16^.

The clinical symptoms and signs of SFTS are mainly non-specific, including high fever, thrombocytopenia, leukocytopenia, diarrhea, hemorrhage, and lymphadenopathy^1^,^3^. A significant portion of patients have shown signs of liver and kidney damage, including elevated serum alanine aminotransferase, aspartate aminotransferase (AST), lactate dehydrogenase (LDH), creatine kinase (CK), and creatine kinase MB fraction (CK-MB) levels^1^,^3^,^17^. The liver may play a central role in the pathogenesis of SFTSV infection as suggested^7^,^18^,^19^. In severe SFTS patients, the clinical conditions may deteriorate rapidly and end in multi-organ dysfunction syndrome, with clinical presentations of central nervous system symptoms and hemorrhagic manifestations. The patients commonly die from disseminated intravascular coagulation (DIC) and multi-organ failure^1^,^3^,^20^,^21^,^22^, of which viral hepatitis may be an important part. A recent report showed that liver lobular necrosis and mild portal fibrosis occurred in SFTS patients and SFTSV antigens could be detected in multiple organs including the liver^18^. In fact the liver may play a central role in the pathogenesis of SFTSV infection as suggested^7^,^18^,^19^, like in infections with other bunyaviruses causing Congo-Crimean Hemorrhagic Fever^23^ and Rift Valley Fever^24^.

Studies on SFTSV infection in mouse and non-human primate models have been reported^13^,^19^,^25^. In a C57/BL6 mouse model, viral RNA and tissue damage were described in spleen, kidney, and liver, but viral replication could only be detected in spleen^19^. However in IFNR-deficient mice, SFTSV replicated in all organs except lungs and viral antigens were detected in multiple organs including the liver^19^. In this report we present novel data from the C57/BL6 mouse model study that previously demonstrated that SFTSV infection induced tissue necrosis responses in the liver^21^. Notably, strong inflammatory responses including cytokine induction were detected in liver tissues. We next demonstrate that SFTSV can infect human liver epithelial cells (HepG2 and MHCC-LM3), in which the virus replicated efficiently. We examined host responses in the infected cells and found that IFN-β signaling was restrained due to the suppression of NSs. Interestingly, our data indicate that NSs activated NFκB signaling, which may be responsible for elevated proinflammatory responses induced predominantly in the liver. We hypothesize that in SFTSV infection, viral NSs induced activation of NFκB in liver cells, which leads to host responses including aberrant cytokine and chemokine induction and increased apoptosis and liver damage.

## Results

### Liver damage and aberrant induction of proinflammatory responses in SFTSV-infected C57/BL6 mice

**Figure 1:**
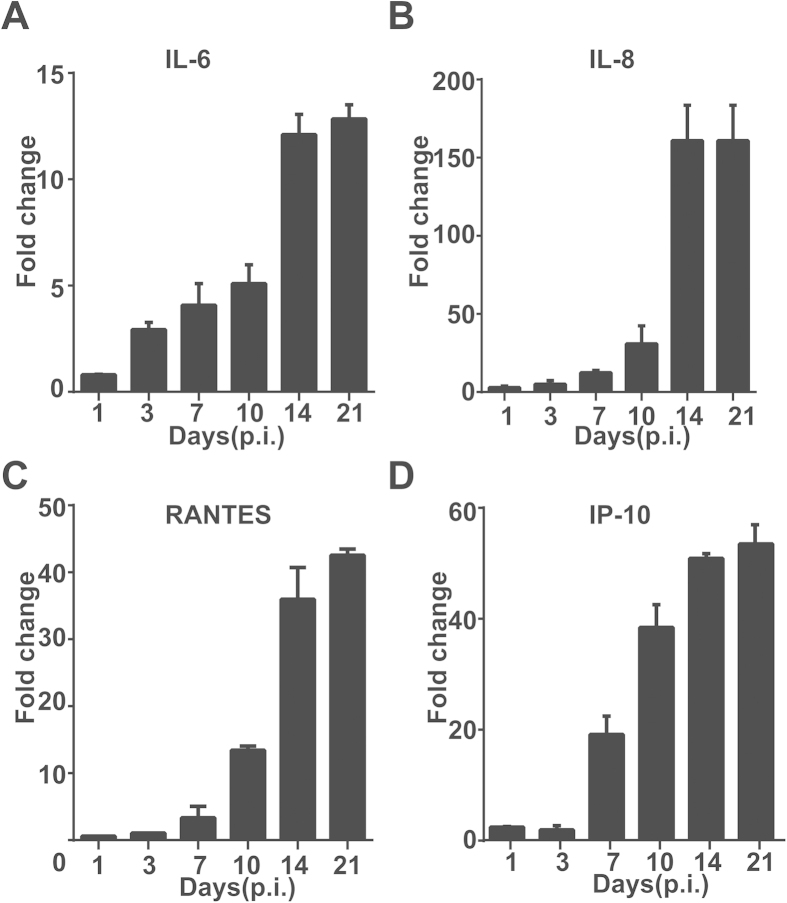
Proinflammatory cytokine and chemokine responses in the liver of SFTSV-infected mice. Mouse infection was conducted at a biosafety level 3 (BSL3) containment in accordance with institutional guidelines. C57/BL6 mice (n = 10) were i.m. inoculated with 10^5^ TCID_50_ SFTSV. Five mice were inoculated with saline and used as parallel controls. At each time point, rectal temperature and weight were measured before the mice were euthanized and liver tissues collected as previously described^17^. Total RNA was extracted after the tissue was ground, and real-time RT-PCR reactions were performed to measure transcript levels of cytokines with specific primers. Fold change for mRNA transcript levels are shown for IL-6 (**A**), IL-8 (**B**), RANTES (**C**), and IP-10 (**D**), and samples were tested in triplicates.

Clinical data in previous studies indicated that varying degrees of liver damage occurred in SFTS patients^1^,^3^,^18^, which may be partly responsible for the multi-organ failures observed in most severe cases. We used a mouse model to study the pathologic changes in liver as well as the whole body by inoculating 6- to 8-week-old C57/BL6 mice intramuscularly (i.m.) with SFTSV. Our previous data showed the serum levels of alanine aminotransferase (ALT) and aspartate aminotransferase (AST) were significantly higher and histopathological changes could be observed in the liver^21^. We extended our study by preparing total RNA from liver tissues of both infected and control mice, collected at different time points post-infection (p.i.), used for realtime RT-PCR analyses with primers specific to mouse cytokines. As shown in Fig. 1, the levels of pro-inflammatory cytokines IL-6 and IL-8 increased at roughly one week p.i. and rose significantly about two weeks p.i. in liver tissues (Fig. 1A,B). A robust induction of chemokines including RANTES (CCL5) and IP-10 was also observed one week after infection (Fig. 1C,D). Taken together, our data indicate that an aberrant regulation of proinflammatory cytokines and chemokines occurred in the liver in infected mice.

### Susceptibility of human liver epithelial cells to SFTSV

**Figure 2:**
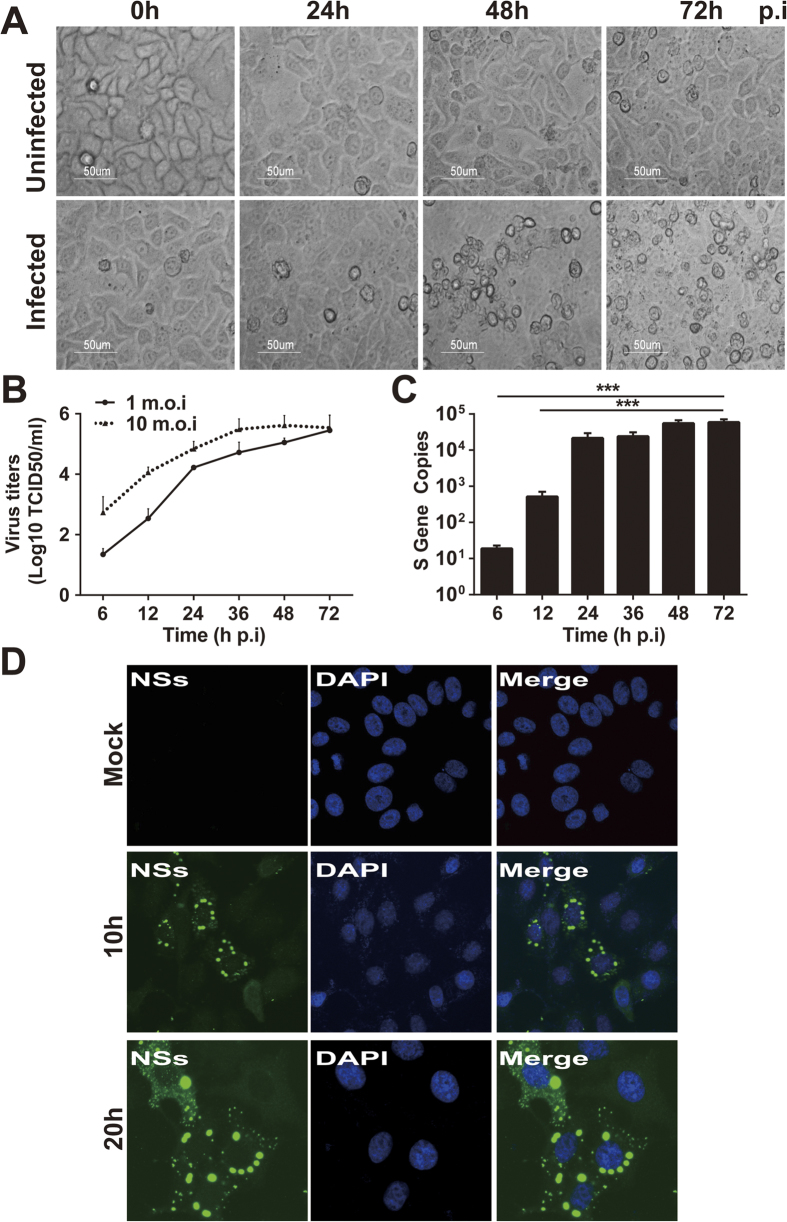
Human liver epithelial cells (HepG2) were susceptible to SFTSV infection. HepG2 cells were mock-infected or infected with SFTSV at an m.o.i of 1. (**A**) Significant CPE was observed in infected cells (magnification 400×) at 48 and 72 hrs p.i. (**B**) Replicative curve of SFTSV in infected HepG2 cells. Culture media of the infected HepG2 cells were taken at various time points p.i., ten-fold serially diluted, and inoculated in Vero cells in 96-well plates. Infectious virus titers were determined after the cells were fixed and stained with an HRP-conjugated anti-viral NP antibody, followed by colorimetric development with TMB substrate. Infection foci were counted and TCID_50_ calculated based on the Reed and Muench method. (**C**) Quantitation of viral RNA in infected HepG2 cells. Total RNA was prepared from uninfected and infected cells at 6, 12, 24, 36, 48, and 72 hrs p.i. and viral RNA were qualified with real-time RT-PCR after reverse transcription. Specific primers for the S gene were used to quantify copy numbers of the S gene. The experiments were repeated at least three times and the data from one representative with two repeats were presented (* p < 0.05). (**D**) SFTSV antigens detected in infected HepG2 cells. Both infected and control cells were fixed at 10 or 20 hrs p.i. After permeablization with PFA, the cells were incubated with rabbit anti-NSs antibody at a dilution of 1:100, followed by staining with Alex Fluro488-conjugated secondary antibody.

To determine how liver cells develop pathogenicity in response to SFTSV, we inoculated human liver epithelial cultures using the human hepatocellular carcinoma cell lines, HepG2 and MHCC-LM3, with SFTSV at an m.o.i. of 1. Typical cytopathic effects (CPEs) appeared at 48 hr and the cell monolayers deteriorated 72 hr p.i. in HepG2 cells (Fig. 2A), demonstrating that SFTSV is able to infect and replicate in human liver epithelial cells. Similar results with cell death were observed with MHCC-LM3 cells (data not shown). We also examined the viral replicative curves as exhibited in Fig. 2B, indicating that SFTSV replicates efficiently in HepG2 with an infectious dose at m.o.i. of either 1 or 10, reaching the same level of about 10^5.5^TCID_50_/ml in culture media at 72 hr p.i. To further confirm efficient viral replication and infection, we extracted total RNA from the cells at various time points p.i. and measured viral S gene copy numbers by realtime RT-PCR. As early as 12 hr p.i., we detected the viral S gene copies at a level of 5.2 × 10^2^, and copy numbers increased to 2.1 × 10^4^ and 5.5 × 10^4^ at 24 and 48 hr p.i., respectively (Fig. 2C).

Infected and control HepG2 cells were also stained with an anti-SFTSV NSs antibody for indirect immunofluorescence staining (Fig. 2D). Viral proteins were detected as early as 10 hr p.i., indicating that human liver epithelial cells were susceptible to SFTSV, which can directly infect and replicate in liver both in human and mice.

### SFTSV induces apoptosis in human liver epithelial cells

**Figure 3:**
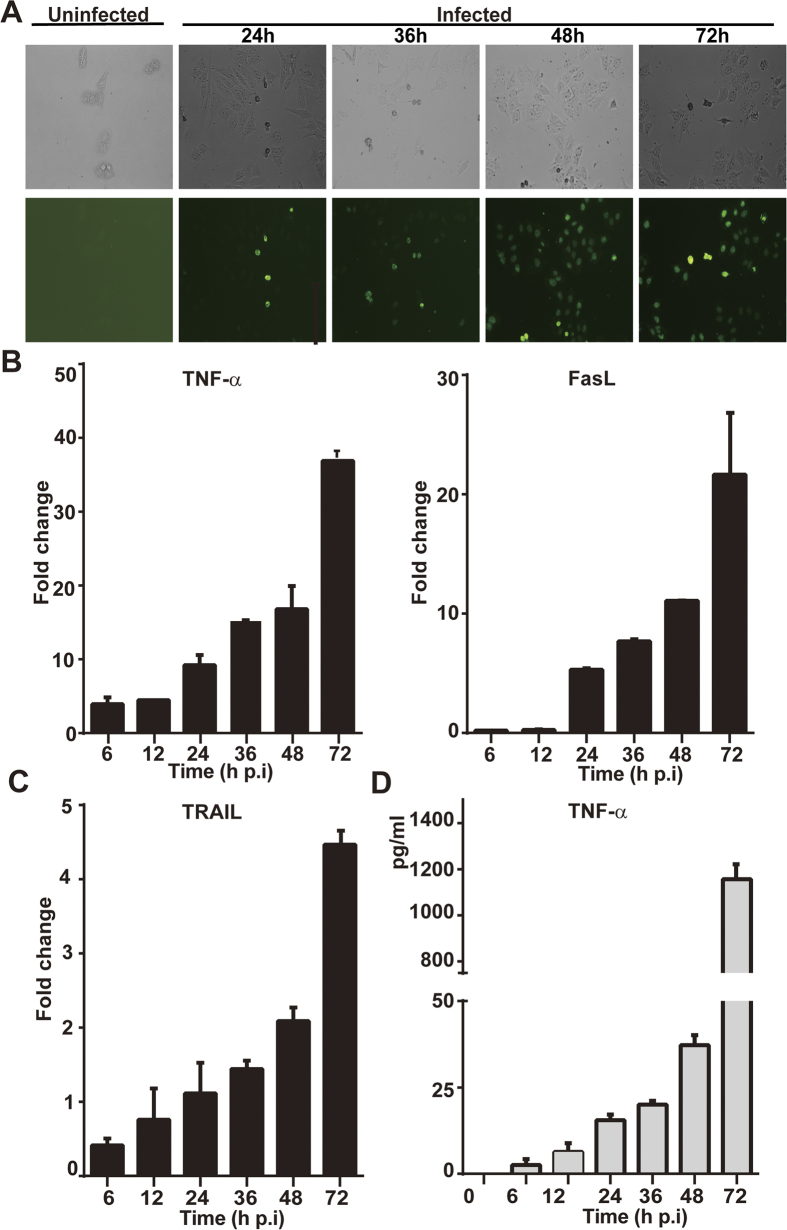
Apoptosis induced by infection of SFTSV in HepG2 cells. (**A)** Cells were infected with SFTSV at an m.o.i of 1. *In situ* apoptosis was detected from 24–72 hrs p.i. by an FITC-dUTP labeled TUNEL assay. (**B**) Transcript levels of TNF-α, FasL, and TRAIL from 6–72 hrs p.i. in infected cells were measured by realtime RT-PCR with specific primers to corresponding cytokines. (**C)** Concentration of TNF-α induced in infected cells. Cultural media of cells infected with SFTSV were collected at various times points p.i. and used for ELISA measurement of the concentration of TNF-α. The assays were repeated at least three times.

We next tried to characterize cell death of liver cells, which may play a role in viral pathogenesis in SFTSV infection, by examining the regulation of death receptor ligands in infected cells by realtime RT-PCR. We found that in HepG2 cells TNF-α and FasL were induced robustly up to 36- and 21-fold at 72 hr p.i. in response to infection (Fig. 3B), but TRAIL remained mainly unchanged (Fig. 3C). While mRNA transcript of TNF-α was upregulated up to 16- and 38-fold at 36 and 72 hr p.i., respectively, TNF-α concentration rose up to 1157 pg/ml in the cultural medium (Fig. 3D).

**Figure 4:**
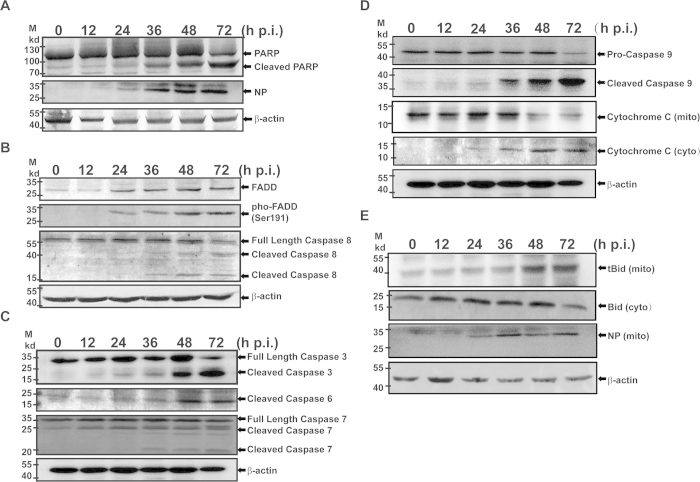
Apoptosis was activated in infected HepG2 cells. Cell lysates were prepared from infected and uninfected cells at indicated time points from 12–72 hrs p.i., and resolved with 10–15% SDS-PAGE. Proteins were transferred to PVDF membranes for western blot analyses with specific antibodies. (**A)** PARP was cleaved in infected cells. (**B**) Upregulation and increased phosphorylation of FADD and increased cleavage and activation of pro-caspase 8. (**C**) Cleavage and activation of executioner pro-caspase −3,−6 and −7. (**D**) Cleavage and activation of pro-caspase −9 and release of cytochrome c from the mitochondrial membrane to the cytosol. (**E**) Cleavage and activation of Bid and translocation of tBid into the mitochondrial membrane.

Both TNF-α and FasL may be critical in SFTSV induced apoptosis through the extrinsic pathway, which was shown with positive TUNEL staining in infected cells (Fig. 3A). The induced apoptosis was confirmed by exhibition of cleaved poly ADP-ribose polymerase (PARP) in cell lysates (Fig. 4A), which increased significantly as SFTSV infection progressed. FasL or TNF-α binding to DR activated pro-caspase 8 cleavage as shown in Fig. 4B. This led to cleavage and activation of downstream executioner caspase −7, –6 and −3 (Fig. 4C), indicating that the extrinsic apoptotic pathway was fully activated. Meanwhile, pro-caspase 9 was cleaved and activated through activation with cytochrome c release from mitochondria to the cytosol as shown in Fig. 4D,E.

Caspase 9/cytochrome c-dependent intrinsic apoptotic pathway in infected HepG2 cells was also apparently activated, probably through activation of Bid to become truncated Bid (tBid), cleaved by active caspase 8. tBid was translocated to the mitochondrial membrane (Fig. 4E), leading to release of cytochrome c into the cytosol for pro-caspase 9 activation. We also observed upregulation of Fas-associated protein with death domain (FADD) and phospho-FADD during the infection, which enhances cleavage and activation of pro-caspase 8. We concluded therefore that both extrinsic and intrinsic apoptotic pathways were involved and tBid may bridge the activation of these two pathways in liver cells infected with SFTSV.

### Pro-inflammatory and innate immune responses are activated in SFTSV-infected liver epithelial cells

**Figure 5:**
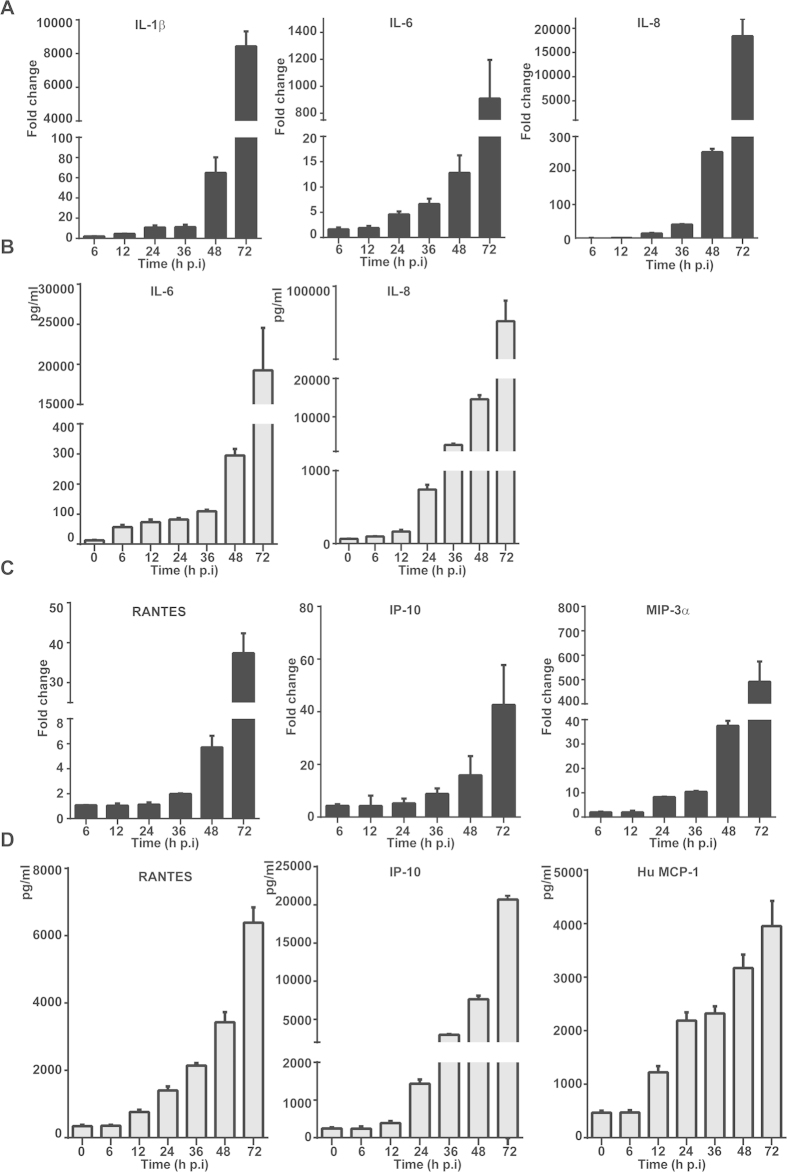
Induction of proinflammatory cytokines and chemokines in SFTSV-infected HepG2 cells. Total RNA was prepared from uninfected or infected cells at indicated time points and used for reverse transcription and real-time PCR analyses with specific primers to cytokine genes. Reactions were conducted in duplicates, each reaction was repeated at least three times; a representative result is presented. Fold changes were calculated relative to a gene in uninfected and infected cells, which were internally normalized to GAPDH. Fold changes in mRNA levels for IL-1β, IL-6 and IL-8 (**A**); and RANTES, IP-10 and MIP-3α **(C)**. Concentrations of the cytokines in the culture media were measured with ELISA for IL-6 and IL-8 (**B**); and CCL5, IP-10 and MCP-1(**D**).

To understand how liver epithelial cells respond to SFTSV, we infected HepG2 cells with the virus at m.o.i. of 1 and examined the transcript expression of proinflammatory cytokines with realtime RT-PCR. RNA transcripts were prepared from infected and non-infected cells collected at different time points p.i. for comparison. We found that the levels of proinflammatory cytokines IL-1β, IL-6, and IL-8 were sharply increased up to 64-, 12- and 254-fold at 48 hr p.i., respectively, and continued to rise up to 8443-, 909- and 18378-fold at 72 hr p.i (Fig. 5A). We confirmed the increase of IL-6 and IL-8 concentration in the cultural medium with ELISA, which showed increases up to 19250 and 84083 pg/ml at 72 hr p.i., respectively (Fig. 5B).

We also observed a robust induction of chemokines such as RANTES (CCL5) and IP-10, which increased up to 37- and 42-fold at 72 hr p.i , and MIP-3α up to 37- and 490- fold at 48 and 72 hr p.i., respectively (Fig. 5C). Correspondingly, the concentrations of RANTES, IP-10, and MCP-1(CCL2) in the cultural medium were significantly increased up to 6387, 20715, and 3957 pg/ml, respectively (Fig. 5D). Similar results with cytokine and chemokine induction in MHCC-LM3 cells infected with SFTSV were also obtained (supplemental Figure S1). Collectively, our data demonstrated clearly that proinflammatory cytokines and chemokines were strongly induced upon SFTSV infection in liver epithelial cells, which may contribute to liver immunopathologenicity.

### Innate immunity and antiviral responses in liver epithelial cells infected with SFTSV

**Figure 6:**
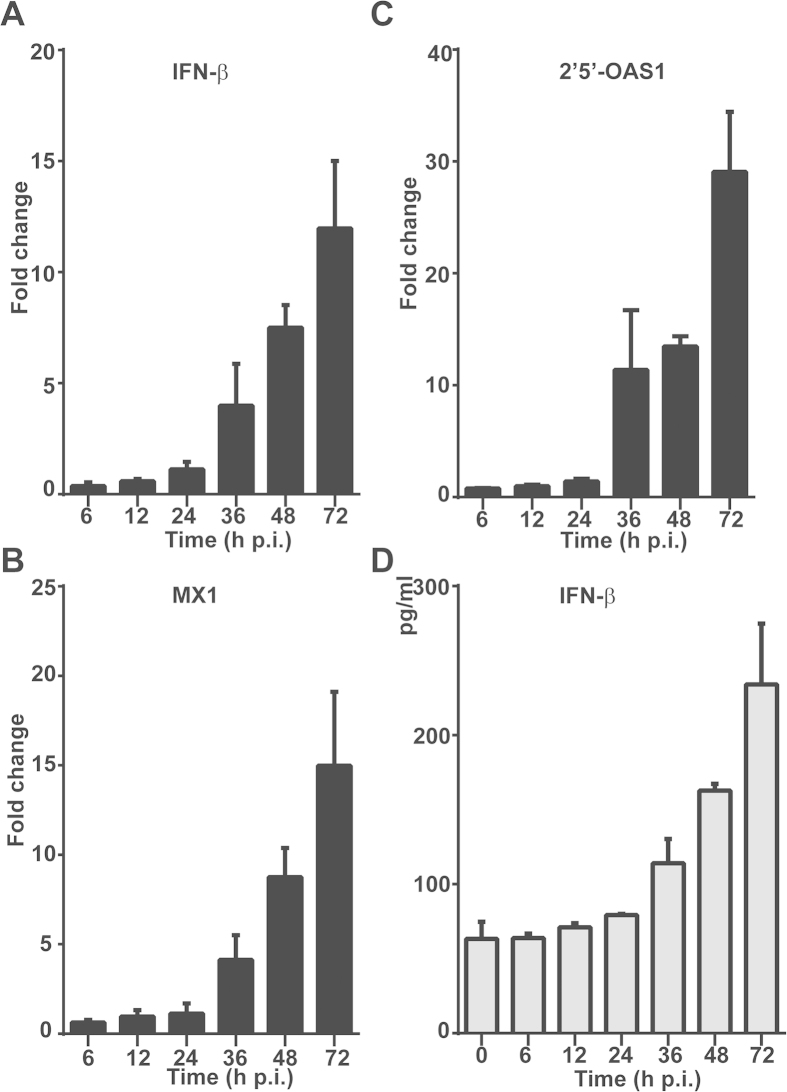
Antiviral responses induced in SFTSV-infected liver epithelial cells. Total RNA was prepared from uninfected and infected cells at various time points p.i., and real-time RT-PCR was performed to measure mRNA transcript levels of IFN-β (**A**), MX1 (**B**), and 2’5’-OAS1 (**C**) at 6, 12 , 24 , 36 ,48 and 72 hrs p.i. respectively. (**D**) Cytokine concentrations in culture media were measure by ELISA for the expression levels of IFN-β.

**Figure 7:**
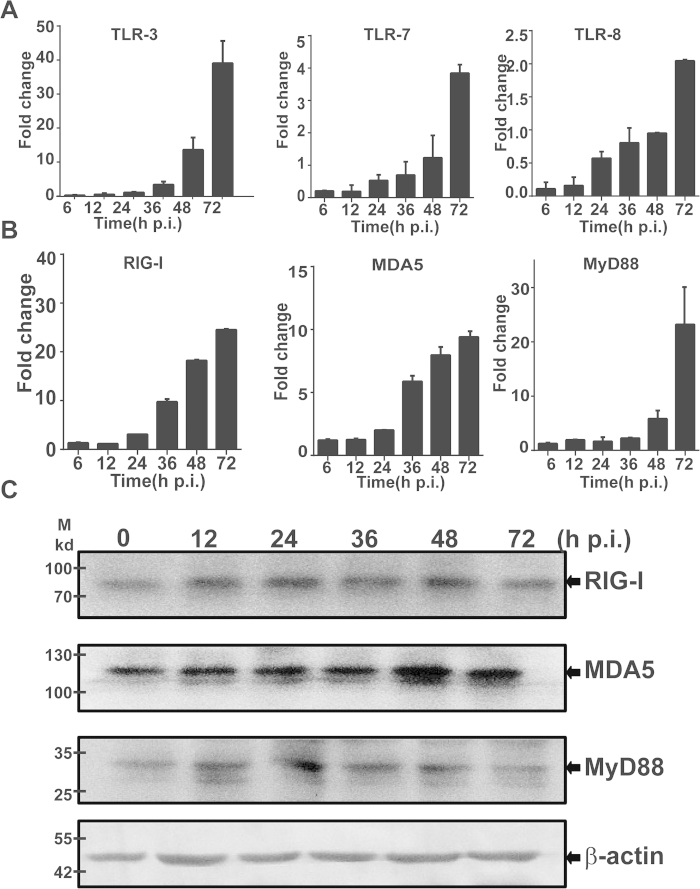
Induction of TLRs and RLRs in SFTSV-infected HepG2 cells. Total RNA was prepared from SFTSV-infected or uninfected cells at different time points and used for reverse transcription and real-time PCR analysis with specific primers to respective genes. Reactions were performed in duplicates, each reaction was repeated for at least three times, and a representative result is presented. Fold changes were calculated relative to a gene in uninfected and infected cells, which were normalized to GAPDH. Fold changes in transcript levels for TLR-3, TLR-7 and TLR-8 (**A**); and MyD88, RIG-I and MDA5 (**B**). Cell lysates were prepared and subjected to SDS-PAGE and western blot analyses with antibodies against RIG-I, MDA-5, MyD88, and β-actin at the time points as indicated.

To assess host antiviral responses in liver cells infected with SFTSV, we examined the induction of antiviral genes, which included IFN-β, IFN-inducible myxovirus resistance 1 (MX1), and 2’,5’-oligoadenylate synthetase 1 (OAS1). Mild induction of mRNA transcripts was detected up to 7.5- and 12-fold for IFN-β, 8- and 15-fold for MX1, and 13- and 28-fold for OAS1 at 48 and 72 hr p.i., respectively (Fig. 6). The concentration of IFN-β was also increased to 162 and 234 pg/ml p.i. These data demonstrate that antiviral host responses were mounted, but the levels were moderate in infected liver epithelial cells. We also examined the induction of toll-like receptors (TLRs) and RIG-I like receptors (RLRs), which are sensors for viral RNA stimulation. Our data indicated that while the levels of TLR-7 and TLR-8 remained almost unchanged after infection, TLR-3 responded significantly up to 13.5- and 39-fold p.i. (Figure. 7A). Upregulation of both RIG-I and MDA-5 was also observed up to 24.5- and 9.4-fold, in addition to the increased expression of MyD88, an adaptor protein in TLR signaling pathway, in HepG2 cells, indicating that the induction of the viral RNA sensors is in general responsive to infection in human liver epithelial cells (Fig. 7B). Increased expression of RIG-I, MDA-5, and MyD88 was also shown in the lysates of the infected cells collected at various time points p.i. by western blot analyses (Fig. 7C).

### Distinct regulation of proinflammatory cytokines by NFκB signal pathway

**Figure 8:**
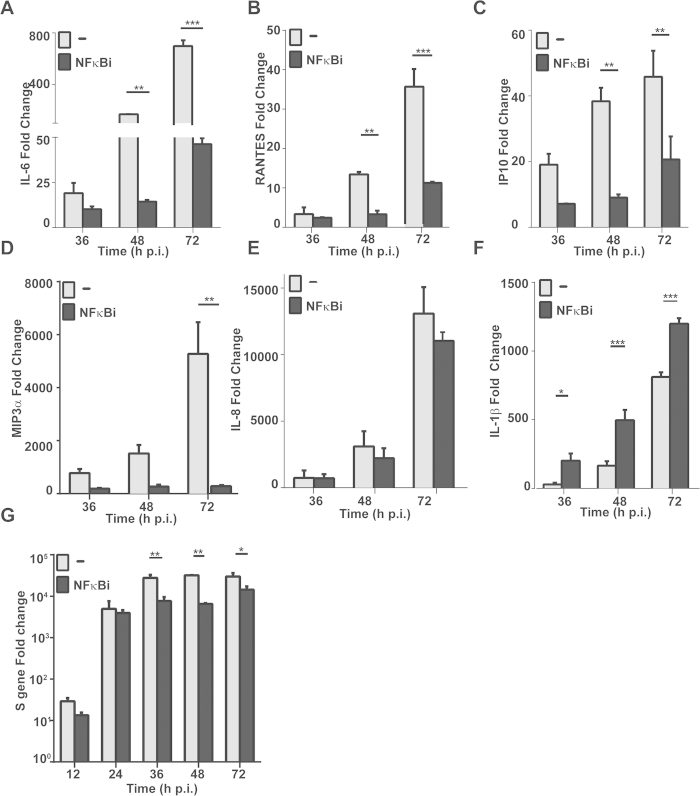
Regulation of proinflammatory cytokine gene transcripts by NFκB. HepG2 cells were pretreated with an NFκB specific inhibitor at 100 nM 1.5 hrs prior to SFTSV infection. Total RNA were prepared at 24, 48, and 72 hrs p.i for reverse transcription. cDNA was used for realtime PCR with specific primers to measure fold changes of cytokine transcripts at different time points. Each assay was repeated at least three times. (**A-F)** Fold change of IL-6, CCL5, IP-10, MIP-3a, IL-8, and IL-1β transcripts. (**G**) Inhibition of viral RNA replication by the inhibitor of NFκB. Realtime RT-PCR with specific primers to the viral S gene was performed to measure the S gene copy numbers in infected cells treated or non-treated with the NFκB inhibitor. Mean fold changes plus standard deviation of the viral S copy numbers were presented from two or three independent assays (*p < 0.05).

To understand how proinflammatory and antiviral cytokines were regulated in SFTSV-infected liver epithelial cells, we examined NFκB signaling and its role in the regulation of the corresponding cytokines. Bay11-7082, an inhibitor of NFκB signaling through preventing phosphorylation and activation of IKKβ and suppressing IKK activity, was used to pre-treat HepG2 cells before viral infection. While no obvious toxicity was observed in the cells treated with Bay11-7082 at the indicated concentrations (data not show), we found significant differences in the induction of the cytokines between untreated and treated cells. The induction of proinflammatory IL-6, RANTES, IP-10, and MIP-3α was significantly suppressed in the presence of the inhibitor (Fig. 8A–D), suggesting that NFκB is responsible for the induction of these cytokines and may play a critical role in immunopathogenicity. On the other hand, the expression of IL-8 was not affected by the treatment of the NFκB inhibitor (Fig. 8E), and the induction of IL-1β was even further increased in the presence of the inhibitor (Fig. 8F), suggesting that the induction of IL-1β occurred in response to SFTSV infection but was somehow suppressed by NFκB signaling in human liver epithelial cells. Meanwhile, our data showed that the S gene copy numbers were significantly decreased in the cells treated with Bay11-7082 (Fig. 8G), compared to those without treatment, suggesting that NF-κB signaling may enhance viral replication in SFTSV-infected cells.

### Differential effect of NSs on IFN-β and NFκB signaling in HepG2

**Figure 9:**
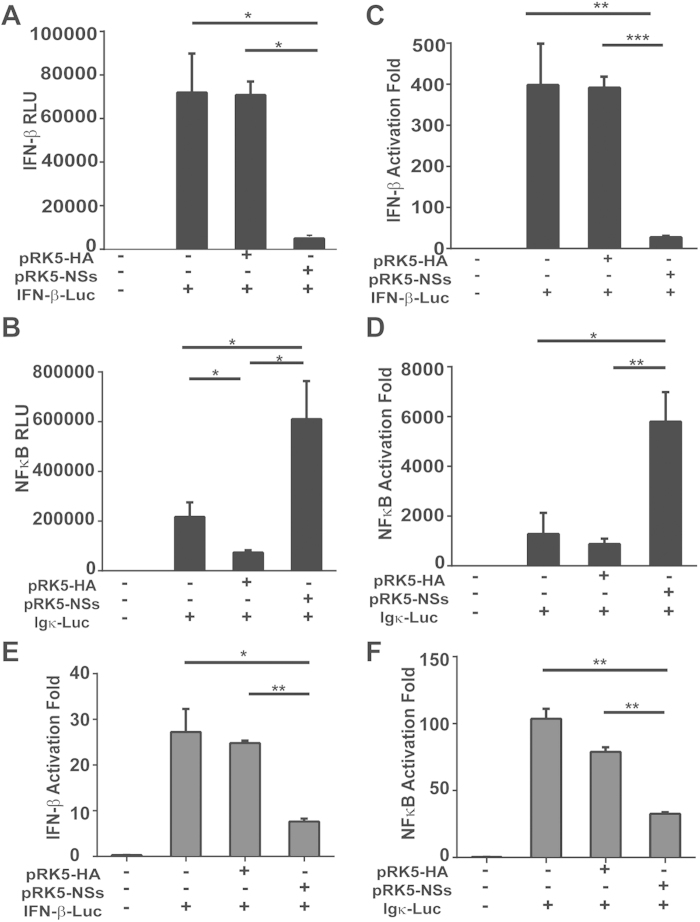
Differential effect of NSs on the activation of NFκB and IFN-β promoter activities. HepG2 (**A**, **B**, **C** and **D**) and HeLa (**E** and **F**) cells were co-transfected with plasmids expressing NSs , pGL3-IFN-β-Luc (**A, C, E**), or pGL3 Ig k-Luc (**B, D, F**) along with pRL8 for 24 hrs , followed by stimulation with 50 μg of poly (I:C)/ml for another 6 hrs. Cell lysates were subjected to lysis and the lysates were measured for luciferase activities. The results are shown as absolute RLU (**A, B**) or activation fold (**C, D**, **E** and **F**).

To assess how NSs, the nonstructural protein of SFTSV, is involved in the modulation of the host responses, we transfected HepG2 cells with a plasmid that expresses NSs transiently^14^,^15^. The cells were co-transfected with a plasmid that either has an IFN-β or NFκB promoter element for a dual luciferase reporter assay. At 24 hr post transfection, the cells were stimulated with 50μg of synthetic poly (I:C), then cell lysates were prepared 6 hr after stimulation and analyzed for luciferase activities. Our results demonstrated that while poly (I:C) enhanced the relative luciferase units (RLU) and activation fold of IFN-β promoter activity up to 7-fold, NSs protein reduced IFN-β promoter activity down to 16% of the original level, indicating that NSs suppressed the IFN-β promoter activation in human liver epithelial cells (Fig. 9A,C). However, a similar assay performed to measure the NFκB promoter activity in NSs-overexpressing HepG2 cells showed that NSs activated the NFκB promoter activity by over three-fold (Fig. 9B,D) in response to poly (I:C) stimulation. This differs from the results in HeLa cells in which NSs inhibited both the IFN-β (Fig. 9E) and NFκB (Fig. 9F) promoter activation. We concluded that viral NSs of SFTSV might facilitate NFκB activation and its target gene expressions in infected liver epithelial cells and promote NFκB-dependent proinflammatory responses in infection.

### Induction of antiviral IFN-β was suppressed by viral NSs

**Figure 10:**
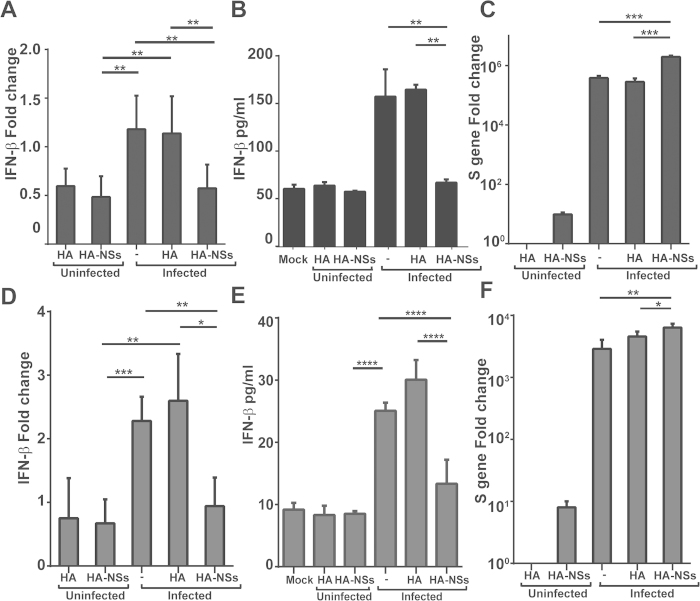
Induction of IFN-β was suppressed by NSs. HepG2 (**A**, **B** and **C**) and HeLa (**D**, **E** and **F**) cells were transfected with cDNA expressing HA-tagged NSs or blank plasmid. After 24 hrs, the cells were mock infected or infected with SFTSV. Total RNA was prepared from non-infected and infected cells at various time points p.i., and real-time RT-PCR was performed to measure the levels of IFN-β (**A** and **D**) and viral S gene copy numbers (**C** and **F**). Cultural media of HepG2 cells were collected at various times points p.i. for ELISA to measure the concentration of IFN-β (**B** and **E**).

We further examined the effect of viral NSs on the induction of antiviral IFN-β and viral replication. Twenty-four hours prior to SFTSV infection, HepG2 and HeLa cells were transfected either with the plasmid expressing NSs or the blanket plasmid. Total RNA was prepared from the cells after SFTSV infection and analyzed with realtime RT-PCR. We also analyzed the IFN-β concentration in the cultural media with or without overexpression of NSs. Our data indicate that induction of IFN-β was suppressed at both RNA and protein levels in the presence of NSs (Fig. 10A,B). Similar results were obtained in HeLa cells as shown in Fig. 10D,E. We also quantified the copy numbers of the viral S gene and detected significant increase of the S gene copy numbers in HepG2 (Fig. 10C) and HeLa cells (Fig. 10F), respectively. Taken together, our data demonstrate that viral NSs may function as a negative modulator of antiviral IFN-β induction and a promoter of viral replication in liver epithelial cells.

## Discussion

SFTS is an emerging febrile illness caused by SFTSV, a novel bunyavirus, presenting with high fever, drastic loss of white blood cells and platelets, and in severe cases, multi-organ failure^1^,^3^,^17^,^20^. Clinically, patients with severe symptoms in SFTSV infection often suffer liver damage, which has not been fully studied. The liver could play an important role in the pathogenesis in SFTSV infection^7^,^18^,^19^, but the detailed mechanism for liver pathology remains unknown. An autopsy report indicated that the liver showed extensive lobular necrosis and mild portal fibrosis and SFTSV NP antigens could be detected in the liver as well as in many other organs. However, the parenchymal cells of each organ including hepatocytes were negative for SFTSV antigens^18^, suggesting that the liver damage may not arise from direct effect of viral infection in hepatocytes. In this report we extended our previous study and confirmed the robust induction of proinflammatory cytokines produced in the liver tissues in C57/BL6 mouse model. We further demonstrated that human liver epithelial cells are susceptible to SFTSV, which replicates efficiently there. The cells underwent apoptosis and produced proinflammatory cytokines significantly in response to SFTSV infection.

Physiologically the liver could be important in viral pathogenesis. Human liver is characterized by a dual blood supply. While 80% of blood enters the liver through the portal vein carrying nutrients from the gastrointestinal tract^22^,^26^,^27^, 20% comes from hepatic artery from the heart pump. The liver is therefore constantly exposed to pathogens or antigenic loads carried from blood or gastrointestinal tract^28^. Ticks biting through skin can transmit SFTSV, and the virus can then penetrate the vascular endothelial membrane into the blood, where it infects and replicates efficiently in blood cells such as monocytes^14^. The liver can be one of the earliest infected organs via penetrating blood cells that harbor the pathogen. Because SFTSV can replicate efficiently in the liver, it might serve as the location for viral amplification before the virus enters the blood with the secondary viremia, therefore playing an important role in viral pathogenesis.

Our data demonstrate the mechanism by which liver damage or viral hepatitis could occur in SFTS patients, especially in those severe cases. In the present study, we show a pattern of host proinflammtory responses in human liver epithelial cells to SFTSV infection. Specifically, cytokines and chemokines including IL-6, IL-8, TNF-α, as well as CCL-5, MIP-3α, and IP-10 were significantly induced, providing an immunopathological basis for liver pathology, deteriorated by infection-induced apoptosis.

Apoptosis is a hallmark event observed upon infection with many viral pathogens, and is executed by a cascade of proteolytic cleavage and activation of cysteinyl proteases and caspases^29^,^30^. Induction of apoptosis was observed in infection with La Crosse virus, a bunyavirus, in BHK and neuroblastoma cells as well as in the brain of newborn mice^31^,^32^. Similar results have been observed in Hantaan^33^, Bunyamwera^34^, Akabane, and Aino viruses in Vero cells^35^, and Punta Toro virus in hamster hepatocytes^32^. Liver damage in SFTSV-infected humans and mice may be attributed to apoptosis induced in liver epithelial cells upon infection as shown in our study, in contrast to the previous observation that SFTSV caused no cell death in many cell types including monocytes, Vero cells, HeLa, and HEK 293 cells^1^,^14^. Apoptosis in HepG2 cells induced by SFTSV was triggered by both extrinsic and intrinsic apoptotic pathways. We have not had evidence to exclude whether autophage was involved in cell death in SFTSV-infected HepG2 cells, and in fact autophagosome-like structure has not been found in SFTSV infection^36^. DR ligands, FasL, and TNF-α were robustly upregulated, binding to DRs and activating a cascade of caspase cleavage and activation including cleavage of pro-caspase 8 and downstream activation of executioner caspase −6, −7 and −3 (Fig. 4B,C). On the other hand, cytochrome c was released from the mitochondria into the cytosol, which subsequently activated pro-caspase −9 (Fig. 4D), an important indicator of apoptosis triggered by the intrinsic pathway^37^, indicating that the mitochondria/cytochrome c-mediated intrinsic apoptotic pathway is also initiated in response to SFTSV infection in human liver epithelial cells. Moreover, we observed that cytosolic Bid was cleaved^38^, probably by active caspase 8^38^,^39^,^40^, and subsequently translocated onto the mitochondrial membrane to promote cytochrome c release into the cytosol to activate pro-caspase 9. Clearly, tBid acts as a bridging initiator between the extrinsic and intrinsic pathways in apoptotic activation in human liver epithelial cells, which may also contribute to hepatic pathology in SFTSV infection.

Proinflammatory responses may be regulated by NFκB signaling pathway in human liver cells. We used an inhibitor of NFκB activation, Bay11-7082, to pre-treat HepG2 cells, followed by SFTSV infection. As our data show, the induction of IL-6, RANTES, IP-10, and MIP-3α, responsive to the viral infection, was significantly suppressed in the presence of the inhibitor (Fig. 8), indicating that these proinflammatory cytokine or chemokine inductions are NFκB-dependent. We noted that the induction of IL-8 was irrelevant to, and the induction of IL-1β may be even suppressed by, NFκB (Fig. 8E,F), indicating that the mechanism of the cytokine regulation is differential, and may be regulated by other pathways such as MAP kinases or PI-3 kinases. It is known that apoptotic responses, including the upregulation of Fas, FasL, or TNF-α, are also regulated by NFκB signaling, but this has not been tested in SFTSV-infected human liver epithelial cells.

We have demonstrated that viral proteins in SFTSV-infected cells can regulate host responses, and that nonstructural NSs plays a key role in the regulation of host antiviral IFN-β signaling^14^,^15^, which has also been observed by other study^36^. NSs protein can interact with TBK1, sequestering TBK1 as well as IKKε and IRF3, components of the IKK complex essential to IFN-β signaling, into NSs-formed viral inclusion bodies in the cytoplasm. The NSs-TBK1 interaction, therefore, prevents translocation of active phosphorylated IRF3 into the nucleus upon viral infection^14^,^15^. Consequently, IFN-β induction is reduced and SFTSV replication enhanced. The data in this study confirm that NSs also suppresses the induction of IFN-β in SFTSV-infected liver epithelial cells (Fig. 9). However, in contrast to its suppressive effect on the NFκB signaling shown in the previous studies, we found that NSs somehow activated the NFκB promoter activity in a luciferase reporter assay (Fig. 9). Considering the role of NFκB signaling in host cytokine and chemokine responses, NSs may be implicated in liver pathology as both a suppressor of antiviral IFN-β and a promoter of proinflammatory responses and immunopathogenicity in infected human liver epithelial cells.

In summary, our study provides evidence to elucidate the mechanism of liver damage, probably as a part of multi-organ failure in severe cases of SFTS, which may result from a combination of virus infection-induced immunopathogenicity, with a hallmark of NFκB-dependent proinflammatory cytokine and chemokine production, and apoptosis triggered by both extrinsic and intrinsic pathways. Our data demonstrate a unique pattern of host responses and mechanisms for their regulation in infected human liver epithelial cells, crucial for further understanding the viral pathogenesis in SFTSV infected humans.

## Methods

### Cells, viruses and reagents

Human liver hepatocellular carcinoma cells (HepG2) were maintained in a mixed medium containing 50% DMEM (Invitrogen, Carlsbad, CA), 50% Ham’s F-12 (Invitrogen), and 10% fetal bovine serum (FBS), with 1% penicillin-streptomycin and 1% L-glutamine at 37°C in an incubator with 5% CO_2_. Another human liver epithelial cell line, MHCC-LM3, obtained from the Liver Cancer Institute, Zhongshan Hospital of Fudan University, Shanghai, African green monkey kidney Vero cells (ATCC CCL-81), and HeLa cells were maintained in DMEM containing 10% FBS, 1% penicillin-streptomycin, and 1% L-glutamine at 37°C.

SFTSV strain HB29^1^, isolated from peripheral blood samples of a patient in Hubei Province, China, was propagated in Vero cells in a BSL-3 laboratory, Chinese Center for Disease Control and Prevention (China CDC), Beijing, and used in this study. All virus aliquots were stored at –80^o^ C. All experiments with the virus and procedures performed in this report have been approved by the Institutional Biosafety Committees (IBC) at Nanjing University and Jiangsu Provincial CDC.

Antibodies including rabbit anti-cytochrome c (sc-7159), goat anti-procaspase-9 (sc-9508), mouse anti-poly (ADP-ribose) polymerase (PARP)(sc-365315), and mouse anti-actin (sc-130300) were obtained from Santa Cruz Biotechnology (Santa Cruz, CA). Antibodies for caspase-3, caspase-7, caspase-8, RIG-I, MDA5, and MyD88 antibodies were purchased from Cell Signaling Technology (Danvers, MA). Rabbit anti-cleaved caspase-6 (sc-9761), anti-cleaved caspase-7 (sc-9491), anti-cleaved caspase-8 (sc-9496), anti-cleaved caspase-9 antibodies (sc-9501), and anti-β-actin antibodies were also obtained from Santa Cruz Biotechnology. NF-κB inhibitor Bay11-7082 (Cat. B5556) was obtained from Sigma-Aldrich (St. Louis, MO).

### Virus infection and titration

The viruses were inoculated in HepG2 cells at a multiplicity of infectivity (m.o.i.) of 1 in DMEM medium free of fetal bovine serum. Following two-hour viral attachment, the inoculum was replaced with the fresh medium containing 2% fetal bovine serum. Cell media were collected at various times points from HepG2 cells infected with the virus for infectious virus titration (50% tissue culture infective dose, TCID_50_). Ten-fold serial dilutions were performed with DMEM to dilute the cultural media, which were subjected to inoculation in Vero cells plated in 96-well plates. Titers of the virus stocks were determined by a previously described TCID_50_ assay^14^ and the stocks were aliquoted and stored at −80^o^ C. Infectious virus titers (TCID_50_/ml) were calculated based on the Reed and Muench method^41^.

### Quantitative real-time PCR

One μg of total RNA extracted from SFTSV- or mock-infected cells with an RNeasy kit (Qiagen, Hilden, Germany) was used for reverse transcription using a Primescript reagent kit (TAKARA, Shiga, Japan). Quantitative real-time PCR was performed with SsoFast^TM^ EvaGreen^®^ Supermix (Bio-Rad Cat.172-5200) following the manufacturer’s instructions. Relative gene expressions were standardized with a glyceraldehyde-3-phosphate dehydrogenase (GAPDH) control. Fold change was calculated according to the formula: 2^(ΔCt of gene - ΔCt of GAPDH)^. For SFTSV S gene, the standard curve was performed with a plasmid containing full-length nucleotides of the S gene according to a previously described protocol^42^. The sequences of oligonucleotide primers used in the study are listed in Table 1. Reactions were implemented in duplicates and repeated three times for each sample, and the mean values and standard deviations were calculated.

### ELISA assay for cytokines

At the same time, from the same group of infected cells, tissue culture supernatants were harvested from control or infected HepG2 cells at indicated time points, followed by centrifugation at 1000 × g for 15 min at 4 °C. A cytokine ELISA kit (Bio-Plex Pro Human 27-plex cytokine panel, Bio-Rad # M50-0KCAF0Y) was used following the manufacturer’s protocol, starting with the preparation of the 8-point standard dilution series and dilution of the samples at 1:4. Fifty μl of beads were added to the assay plate for incubation in the dark at room temperature (RT) with shaking at 300 rpm for 30 min, followed by subsequent washes. After incubation with 25 μl of biotin-conjugated detection antibody, 50 μl of streptavidin-phycoerythrin (SA-PE) was added. Following the final addition of 125μl assay buffer and shaking at 1,100 rpm for 30 sec, the plates were read and data analyzed with Bio-Plex manager software. The assay was repeated twice.

### Terminal transferase-dUTP nick end labeling (TUNEL)

Non-infected and infected HepG2 cells were fixed with 4% paraformaldehyde (PFA) at RT for 30 min and permeabilized with 0.1% Triton X-100 on ice for 10 min. A TUNEL staining using an *in situ* cell death detection kit (Roche, Indianapolis, IN) was performed on infected or mock-infected cells with FITC-conjugated dUTP labeling according to manufacturer’s instructions. The stained slides were observed under a Nikon inverted fluorescence microscope.

### Subcellular protein extraction and western blot analysis

Cell lysates were prepared by lysis of uninfected and infected HepG2 cells in 1% NP-40 lysis buffer containing 10 mM HEPES (pH 7.9), 1.5 mM MgCl_2_, 10 mM KCl, 0.5mM DTT, 2 mM PMSF, 2 mM NaF, 1 mM Na_3_VO_4_, 1 μg/ml aprotinin, and 1 μg/ml leupeptin on ice for 20 min. Supernatants were harvested as the cytosolic fraction after centrifugation (500 g, 5 min at 4 ^o^C). For the mitochondrial fraction, we performed the preparation using KaiJi mitochondrial protein extraction kit (Keygentec, Nanjing, China) following the provider’s instructions. The resultant lysates were separated by SDS-PAGE and the proteins transferred to Immuno-Blot PVDF membrane (Millipore, Billerica, MA). The membrane was blocked with TBS-Tween 20 (TBST) containing 5% nonfat milk for 40 min at RT and incubated with appropriate primary antibodies diluted in TBST at 4 ^o^C overnight. After incubation with primary antibodies, the membrane was washed three times with TBST, followed by further incubation with alkaline phosphatase (AP)-conjugated anti-rabbit, anti-mouse, or anti-goat IgG antibodies (Sigma) for 1.5 hr at RT. After three washes, BCIP/NBT reagents (Invitrogen) were used for colorimetric development. β-actin levels were detected as input controls in each experiment.

### Immunofluorescence analysis

SFTSV-infected and uninfected HepG2 cells were fixed with 4% paraformaldehyde (PFA) at RT for 30 min and permeablized with 0.1% Triton X-100 on ice for 10 min, followed by three washes with PBS, then blocked with 5% BSA at 37 ^o^C for two hr. The cells were incubated with a rabbit anti-SFTSV nonstructural protein NSs antibody^16^ at 1:100 dilution in PBS-Tween (PBST) containing 1% BSA at 4 ^o^C overnight. After three washes with PBST, the cells were incubated with FITC-conjugated anti-rabbit antibody at 1:200 dilution at 37 ^o^C for one hr. The cells were washed three times with PBST and incubated with 1 μg/ml DAPI in PBS for 5 min. After three washes with PBST, the cells were covered with one droplet of anti-fade reagent (Sigma) and observed under an Olympus laser scanning confocal microscope.

### Dual-luciferase Reporter Assay for IFNβ and NFκB promoter activity

HepG2 cells were seeded in 24-well plates at a density of 2.5 × 10^5^ cells per well. The next day, they were transfected with blanket pRK5 plasmid or pRK5 expressing NSs as described previously^14^,^15^, along with pGL3-IFNβ-Luc or pGL3-Igκ-Luc, respectively, and pRL8-SV40 using Lipofectamine 2000. Total amount of DNA was kept identical in each transfection by adding blanket control plasmid. At 24 hr after transfection, the cells were stimulated with 50 μg/ml poly (I:C) for 6 hr, and cell lysates were prepared 24 hr later and used to determine Firefly and Renilla luciferase activities (Promega, Madison, WI) according to the manufacturer’s instructions.

### SFTSV infection in C57/BL6 mice

As described previously^21^, The SFTSV infectious animal experiments were conducted under biosafety level 3 (BSL3) containment in accordance with institutional guidelines. C57/BL6 mice were inoculated i.m. with 10^5^ TCID_50_ of SFTSV. Five mice were injected with saline and used in parallel as controls. At each time point, ten mice in each group were sacrificed and mouse liver tissues were collected for tissue RNA extraction. The SFTSV challenge experiments in mice were conducted under biosafety level 3 (BSL-3) containment, in accordance with China CDC’s institutional guidelines on animal use.

### Statistical analysis

For statistical analysis, a two-tailed Student’s t-test was used to evaluate realtime RT-PCR data. An *x*^2^ analysis was used to evaluate significant differences of the data in two and more groups. The 0.05 level of probability (p < 0.05) was considered statistically significant.

## Additional Information

**How to cite this article**: Sun, Q. *et al.* Host Responses and Regulation by NFκB Signaling in the Liver and Liver Epithelial Cells Infected with A Novel Tick-borne Bunyavirus. *Sci. Rep.* **5**, 11816; doi: 10.1038/srep11816 (2015).

## Supplementary Material

Supplementary information

## Figures and Tables

**Table 1:** Sequences of the primers used for detecting genes by real-time PCR.

Genes	Primer 5’	Primer 3’
S(HB29)	GGGTCCCTGAAGGAGTTGTAAA	TGCCTTCACCAAGACTATCAATGT
IL-1β	GGACAAGCTGAGGAAGATGC	TCGTTATCCCATGTGTCGAA
IL-6	AAAGAGGCACTGGCAGAAAA	TTTCACCAGGCAAGTCTCCT
IL-8 IP-10	GTTCCACTGTGCCTTGGTTT	GCTTCCACATGTCCTCACAA
RANTES	GAATCGAAGGCCATCAAGAA	CCTCTGTGTGGTCCATCCTT
IFN-β	TACACCAGTGGCAAGTGCTC	TGTACTCCCGAACCCATTTC
MX1	TGCTCTGGCACAACAGGTAG	CAGGAGAGCAATTTGGAGGA
OAS1	ACCACAGAGGCTCTCAGCAT	CTCAGCTGGTCCTGGATCTC
MIP-3α	CGATCCCAGGAGGTATCAGA	TCCAGTCCTCTTCTGCCTGT
TLR-3	TTTATTGTGGGCTTCACACG	GATTTGCGCACACAGACAAC
TLR-7	AGCCTTCAACGACTGATGCT	TTACGAAGAGGCTGGAATGG
TLR-8	TACAGGAAGTTCCCCAAACG	ATTTTGCAGCCCTTGAAATG
MyD88	GCTCATCGAAAAGAGGTTGG	ACATTCCTTGCTCTGCAGGT
RIG-I	AGATTTTCCGCCTTGGCTAT	ACTCACTTGGAGGAGCCAGA
MDA5	CAGGGAGTGGAAAAACCAGA	TTTCCAGGCTCAGATGCTTT
FasL	GGCCTGTGTCTCCTTGTGAT	TGCCAGCTCCTTCTGTAGGT
TNF-α	GGAGTTTGATGGCAACCAGT	TCTCCTCCTGCATCACACAG
GAPDH	ACAGTCAGCCGCATCTTCTT	ACGACCAAATCCGTTGACTC
