# Supplementary material for: Host Responses and Regulation by NFκB Signaling in the Liver and Liver Epithelial Cells Infected with A Novel Tick-borne Bunyavirus
Source: Sci Rep. 2015 Jul 2;5:11816. doi: 10.1038/srep11816 (PMC4488873; doi:10.1038/srep11816)

Supplemental Materials: SREP-15-01164A

Host Responses and Regulation by NFκB Signaling in the Liver and Liver Epithelial Cells Infected with the Novel Tick-borne Bunyavirus

Qiyu Sun[1], Cong Jin[2], Lili Zhu[1], Mifang Liang[2], Chuan Li[2], Carol J. Cardona[3], Dexin Li[2]*, and Zheng Xing[1,3]*

1 The Key Laboratory of Pharmaceutical Biotechnology and Medical School, Nanjing University, Nanjing, China.
2 National Institute for Viral Disease Control and Prevention, Chinese Center for Disease Control and Prevention, Beijing, China.
3 Veterinary and Biomedical Sciences, College of Veterinary Medicine, University of Minnesota, Twin Cities, Saint Paul, MN, USA.

*Corresponding authors:

Zheng Xing, Mailing address: 300D Veterinary Science Building, University of Minnesota at Twin Cities, 1971 Commonwealth Avenue, Saint Paul, MN 55108.

E-mail: zxing@umn.edu Tel: (612) 626-5392 Fax: (612) 626-5203

or Dexin Li, Mailing address: National Institute for Viral Disease Control and Prevention, Chinese Center for Disease Control and Prevention, Beijing, China.

E-mail: lidx@chinacdc.cn

Supplemental Figure F1. Induction of proinflammatory cytokines and chemokines in SFTSV-infected MHCC-LM3 cells. Total RNA was prepared from uninfected or infected cells at indicated time points and used for reverse transcription and real-time PCR analyses with specific primers to cytokine genes or the S gene. Reactions were conducted in duplicates and each reaction was repeated at least three times; a representative result is presented. Fold changes were calculated relative to a gene in uninfected and infected cells, which were internally normalized to GAPDH. Fold changes in mRNA levels for IL-1β(**A**), IL-6(**B**), IL-8 (**C**) and MIP-3α **(D)**; and RANTES(**E**), IP-10(**F**), TNF-α **(G)** and S gene **(H)**.

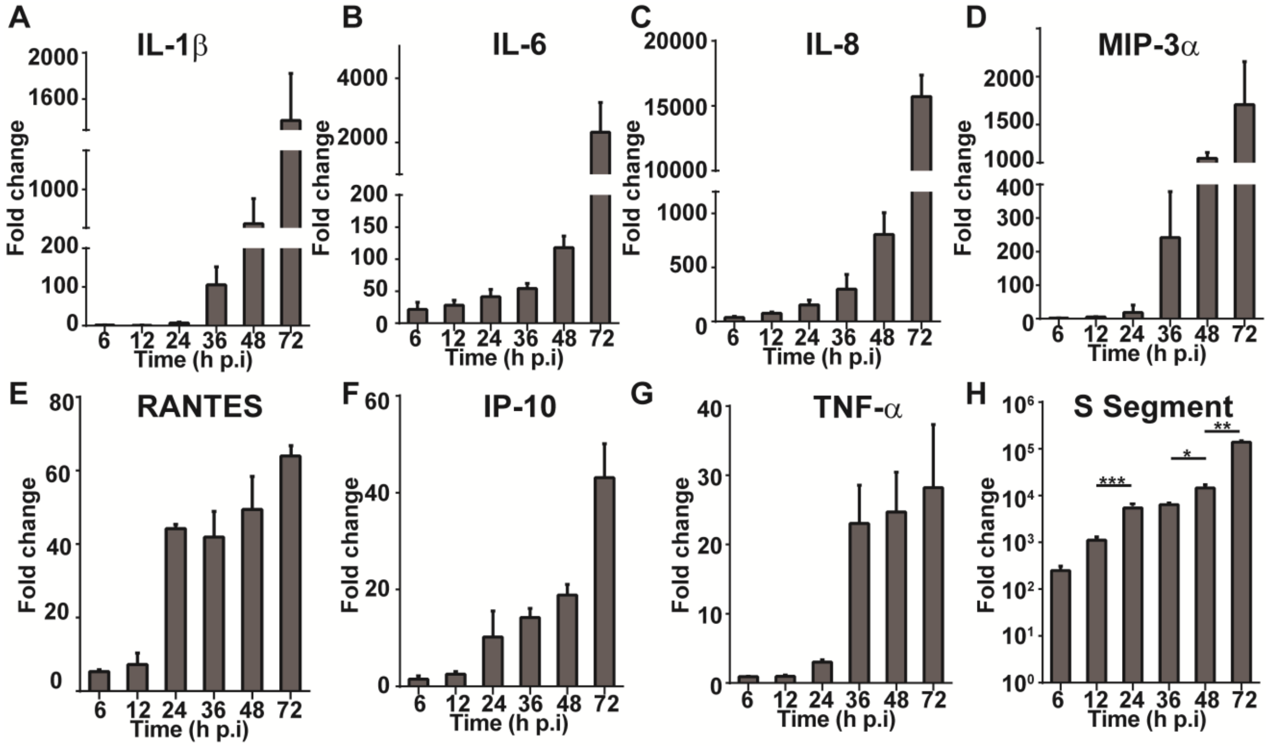

Supplement: Supplementary information [file srep11816-s1.pdf]
